# Paraspinal Skip Lesion Encountered During the Resection of a Sacral Chordoma: A Case Report

**DOI:** 10.7759/cureus.103022

**Published:** 2026-02-05

**Authors:** Ian K Christman, Jason Ina, Jacob Speybroeck, Shahrazad Saab, Brandon Jonard

**Affiliations:** 1 Orthopaedic Surgery, University Hospitals Cleveland Medical Center, Cleveland, USA; 2 Pathology, University Hospitals Cleveland Medical Center, Cleveland, USA

**Keywords:** chordoma, paraspinal lesion, sacral chordoma, sacrectomy, skip lesion

## Abstract

We present the case of an 88-year-old man who underwent a resection of a sacral chordoma. During the resection, a paraspinal skip lesion was encountered. Ultimately, resection of the sacral chordoma was completed with no adjuvant radiation, and at 30 months postoperatively, there are no signs of metastasis or local recurrence. This case represents an unusual case of discontinuous skip lesions in the paraspinal muscles encountered during chordoma resection. In this instance, resection without adjuvant therapy was successful in preventing recurrence. Postoperatively, the patient should be monitored for metastasis and local recurrence with imaging locally as well as of the chest per institutional protocols.

## Introduction

Sacral chordomas, though rare, are the most common primary malignant neoplasm of the sacrum [1]. The incidence of chordomas is 0.8 in 1,000,000 people per year and usually presents during the fifth or sixth decade of life at advanced stages due to their slow-growing and indolent nature [2-5]. Many times, they present with vague symptoms such as lumbar or gluteal pain. Other symptoms can include radiculopathy and bowel or bladder dysfunction [1]. Histologically, sacral chordomas exhibit vacuolated cells encased in a myxoid matrix with mild nuclear atypia [3,4]. 

The gold standard for the treatment of sacral chordomas is complete en bloc resection with negative margins [2,3,6-9]. Common complications of resection are caused by damage or resection of local nerves and nerve roots and include lower limb weakness, bowel/bladder incontinence, and sexual dysfunction [1,4,7,8,10]. Historically, the effectiveness of radiation therapy has been debated, though newer data support the use of hadron therapy (proton or carbon ions) as adjuvant or standalone treatment [1,3-6,8,9,11]. Sacral chordomas are relatively resistant to chemotherapy though there have been recent advances in targeted molecular therapies [2,3,5].

Even though chordomas are slow-growing, 5-40% of patients will develop metastasis, and 43-85% will develop local recurrence [2,12]. The most common sites of metastasis are the lungs, bones, skin, liver, and lymph nodes [2,6]. Locally, the tumor can invade the sacroiliac joints, the sacral nerve roots, as well as the musculature of the hips and pelvis [2]. Sacral chordomas are surrounded by a pseudocapsule that can contain tumor cells which can contribute to local recurrence [1,2]. Survival is reported to be 67.6% and 39.9% at five and 10 years, respectively [4]. Patients can also develop skip lesions with sacral chordomas, which are tumor cells in surrounding tissues distinct in location from the main tumor lesion but are chordomas histologically.

We present the case of an 88-year-old man who underwent resection of a sacral chordoma. During the operation, an intraoperative biopsy-proven paraspinal skip lesion that was separate from the main sacral mass was encountered and resected. Skip lesions have been described primarily in gluteal musculature [13], but to our knowledge, there is no literature available for a sacral chordoma with paraspinal skip lesions. 

## Case presentation

**Figure 1:**
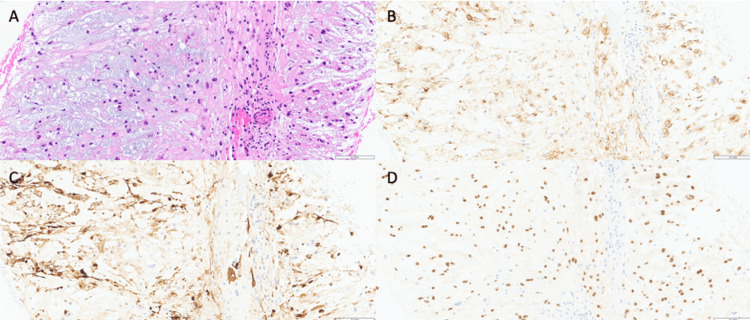
Core needle biopsy of the sacral chordoma Hematoxylin and eosin stain (A) of the biopsy sample demonstrating physaliphorous cells arranged in cords within a myxoid stroma and separated by fibrovascular septa with strong immunohistochemical expression of S100, EMA, and Brachyury (B, C, and D).

An 88-year-old man with a past medical history significant for monoclonal gammopathy of undetermined significance (MGUS) underwent a positron emission tomography (PET) scan for routine surveillance which revealed a hypermetabolic sacral lesion five months prior to his initial presentation to the orthopaedic oncologist. A core needle biopsy was performed and demonstrated a lobulated, epithelioid tumor comprised of large cells with clear, bubbly cytoplasm (physaliphorous cells) in a myxoid stroma. Tumor lobules were separated by fibrovascular septa. Tumor cells demonstrated strong immunohistochemical expression of S100, EMA, and Brachyury, the latter being the hallmark of chordoma as seen in Figure 1.

**Figure 2:**
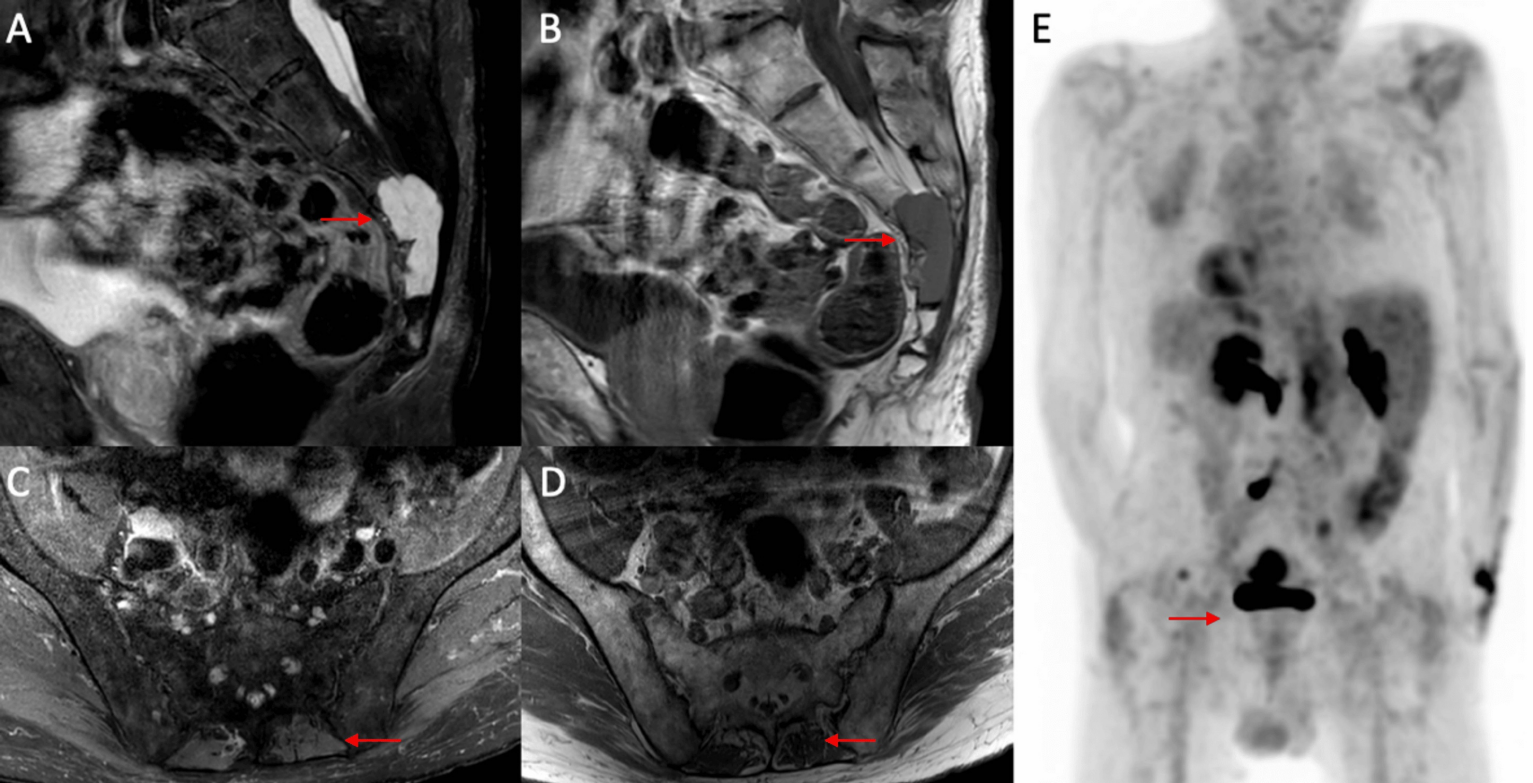
MRI of the sacral chordoma and PET scan Sagittal T2-weighted (A) and T1-weighted (B) MRI of the pelvis demonstrating a 4×2.8×1.5 cm sacral lesion from S3 to S5. Axial T2-weighted (C) and T1-weighted (D) MRI of the pelvis at S2 demonstrating no appreciable lesion in the left paraspinal musculature preoperatively. Posterior view of the preoperative PET scan demonstrating no appreciable lesion in the left paraspinal musculature (E). MRI: magnetic resonance imaging; PET: positron emission tomography

The findings are diagnostic of conventional chordoma. A PET scan seen in Figure 2 did not reveal any metastatic lesions.

On initial presentation, the patient had been asymptomatic without any lower extremity weakness or bladder or bowel complaints. Magnetic resonance imaging (MRI) demonstrated a T2 hyperintense lesion with contrast enhancement involving S3-S5 with a 4×2.8×1.5 cm tissue mass extending posteriorly seen in Figure 2. No paraspinal lesions were seen on MRI.

There was no invasion anteriorly with a fat plane present between the rectum and sacrum. Non-operative treatment options in the form of proton therapy were discussed with the patient. Operative treatment offered included wide excision via a partial sacrectomy. Based on the location of the tumor, it was thought that the S1-S3 nerve roots could be preserved. After a detailed discussion about treatment options, the patient chose to proceed with surgical management.

**Figure 3:**
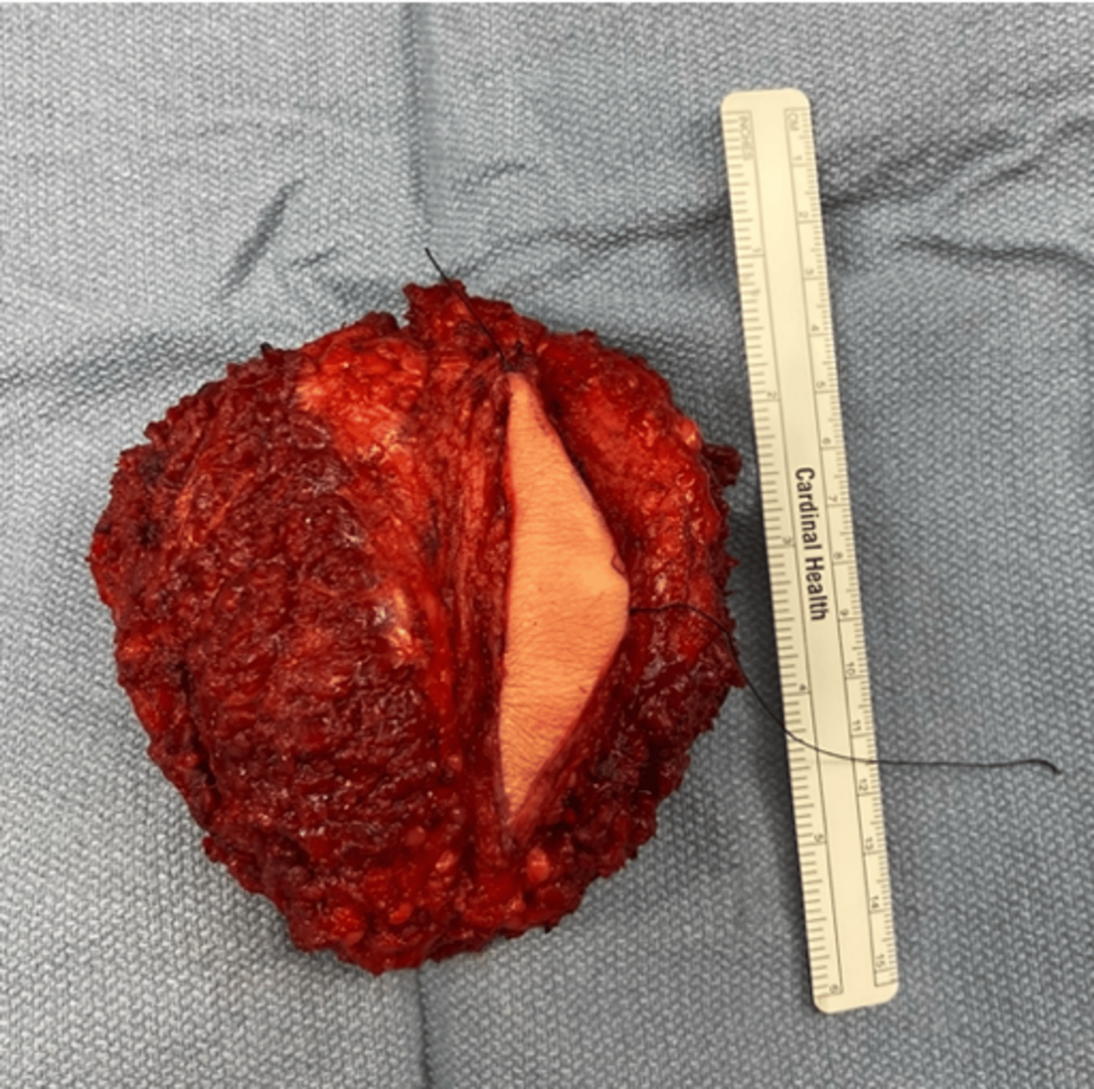
Resection specimen of the sacral chordoma En bloc resection specimen of the sacrum from S3 to S5 with surrounding tissue. The tissue contains S4 and S5 nerve roots.

Six months after the initial PET scan, the patient was brought to the operating room for wide excision of the sacral chordoma. A standard posterior approach to the sacrum was utilized starting at the S2-S3 transition. While dissecting the paraspinal musculature of the spinous processes, a 0.5 cm nodule within the left paraspinal musculature at the level of S2 was noted near the sacrum but not coming off of the bone. The nodule was sent off as a frozen section to pathology and was consistent with chordoma. There was no evidence of expansion of the mass in the field of view or on preoperative imaging so the nodule was believed to be a skip lesion. After completing the release of musculature off the lamina, the S2 and S3 nerve roots were identified and decompressed via laminectomy. The S4 and S5 nerve roots were ligated and transected. An osteotomy was then performed starting at the body of S3, and the sacrum was resected caudally and was sent to pathology as a permanent specimen seen in Figure 3 with resultant negative margins.

**Figure 4:**
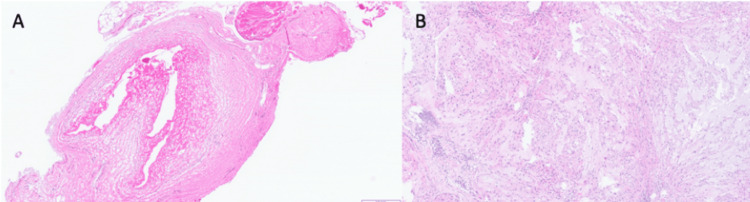
Biopsy of the skip lesion Hematoxylin and eosin stain of the skip lesion (A) showing a tumor nodule histologically similar to the resection specimen (B).

Intraoperative frozen section at the level of the osteotomy was negative for tumor. Plastic surgery then assisted with the complex closure with a gluteus maximus rotational flap. Postoperatively, the patient's hospital course was complicated by respiratory depression which required an extended stay in the ICU. The patient also had bowel and bladder incontinence postoperatively with close follow-up with both urology and general surgery. He was discharged from the hospital on postoperative day 34. Final pathology on the resection specimen and the skip lesion was conventional chordoma, and both demonstrated similar histology as seen in Figure 4.

The patient returned to the clinic two months postoperatively for his first follow-up visit with persistent bowel and bladder incontinence. After discussion with radiation oncology and the family, it was determined that even though there may be an increased likelihood for recurrence due to the skip lesion, it would be best not to move forward with radiation due to the large surgical field, the patient's age, and the past complication of incontinence. The plan would be close monitoring with MRI and use radiation to any areas where recurrence may be indicated. Follow-up MRI at six, 12, 18, 24, and 30 months postoperatively demonstrated no signs of local recurrence. Chest X-ray at each visit and chest computed tomography (CT) at 12 months demonstrated no signs of pulmonary metastasis. The patient had persistent bladder incontinence requiring a suprapubic catheter but reported improvement in bowel incontinence. Additional follow-up will include chest X-ray every six months and pelvic MRI annually to monitor for local recurrence and metastasis. 

## Discussion

Our patient was diagnosed with a sacral chordoma found incidentally on a surveillance PET scan for MGUS with no presenting symptoms. During resection of the sacral chordoma, a skip lesion was found in the paraspinal musculature that was consistent with sacral chordoma on pathology. The skip lesion was not seen on prior imaging and was in a different location than the biopsy tract, ruling out tumor recurrence along the tract. After discussion with the family, resection was continued even with the potential for increased risk of local recurrence with the presence of the paraspinal skip lesion. The S1-S3 nerve roots were able to be preserved during the resection, and negative margins were obtained.

Postoperatively, the patient developed urinary and bladder incontinence and ultimately elected to have placement of a suprapubic catheter. He did, however, report partial improvement in bowel incontinence with time. He did not experience any lower extremity weakness and was able to ambulate without difficulty. Adjuvant proton beam radiation was deferred due to the patient's age, the large surgical field, and the past complications of incontinence. He continued to have routine follow-up with pelvic MRI and chest CT to monitor for local recurrence and metastasis. At 30 months follow-up, there were no signs of local recurrence or metastasis.

When managing a sacral chordoma, it is important to consider patient-specific factors in treatment. En bloc resection with negative margins is the optimal management for patients. Ruggieri et al. studied 56 patients with a sacral chordoma and found a significant decrease in local recurrence in patients who underwent wide resection compared to those who underwent marginal or intralesional resection [12]. Radiation therapy alone can be considered in patients with risk factors for surgery, elderly patients, or patients who do not want to experience the potential side effects of the surgery. Postoperatively, we offer adjuvant radiation therapy with protons for local control in cases of incomplete resection. Chhabra et al. studied 100 patients with chordomas who underwent proton therapy with a local control rate of 97% and 94% at two and three years, respectively [11]. However, in situations such as the presented case, it may be optimal to forgo radiation therapy due to the patient's age, size of resection, comorbidities, and other factors. During resection, an attempt should be made to obtain negative margins while preserving as many sacral nerve roots as possible. Todd et al. studied 53 patients who underwent a sacrectomy and found that 100% of patients with preserved bilateral S3 roots had retained normal bowel function and 69% had retained normal bladder function [14]. Even with the preservation of S3 bilaterally, our patient experienced bowel and bladder incontinence. It is imperative that prior to surgery, patients are counseled on potential complications including bowel and bladder incontinence, lower extremity motor weakness and sensory loss, and sexual dysfunction [1,4,7,8,10]. After surgery, it is important to maintain routine follow-up with pelvic MRI and chest CT to monitor for local recurrence and metastasis with the literature reporting a local recurrence rate of 43-85% and a metastasis rate of 5-40% [2,12]. Furthermore, it is necessary to manage sacral chordomas with a multidisciplinary team many times including orthopaedic surgery, radiation oncology, plastic surgery, and medical oncology for optimal results. 

The only other study we found in the literature that studied skip lesions associated with chordomas was Akiyama et al. They followed 40 patients who underwent en bloc sacral chordoma resections, 17 of which were found to have skip lesions, all located in the gluteus maximus. They concluded that the presence of skip lesions was associated with an increased risk of local recurrence [13].

## Conclusions

Based on the results of this case report, we believe that the presence of paraspinal skip lesions intraoperatively should not preclude the continuation of a planned resection of a chordoma with the additional resection of identified skip lesions. It is important to counsel the patient postoperatively that the risk of local recurrence may be increased due to the presence of these skip lesions and they will require routine follow-up with pelvic MRI and chest X-rays and CT scans for the monitoring of local recurrence and metastasis, respectively. We recommend adjuvant radiation therapy with protons if the patient is amenable, but in our case, the patient forwent radiation and continued with routine monitoring as he was not a suitable candidate to undergo radiation therapy. 
